# Clinical Outcomes and Re-Splinting in Pediatric Dental Trauma Managed with Titanium Trauma Splints: Insights from a Hospital-Based Retrospective Study

**DOI:** 10.3390/medicina62061146

**Published:** 2026-06-12

**Authors:** Elvira Ferrés-Amat, Sira Herrera-Martínez, Cristina Díaz-Martínez, Isabel Maura-Solivellas, Maria Jesus Campillay, Iván Valdivia-Gandur, Eduard Ferrés-Padró

**Affiliations:** 1 Service of Pediatric Dentistry, Hospital HM Nens, HM Hospitales, 08009 Barcelona, Spain; elvira.fa@institutferresamat.com (E.F.-A.); sira.herrera@hotmail.com (S.H.-M.); mcristina_diaz@hotmail.com (C.D.-M.); ismaurasol@hotmail.com (I.M.-S.); 2 Pediatric Dentistry Department, Faculty of Dentistry, Universitat Internacional de Catalunya (UIC), 08195 Barcelona, Spain; 3 Pediatric Dentistry Service, Institut Ferrés Amat, 08021 Barcelona, Spain; 4 Service of Oral and Maxillofacial Surgery, Hospital HM Nens, HM Hospitales, 08009 Barcelona, Spain; eduard.fp@institutferresamat.com; 5 Instituto de Investigación Sanitaria, HM Hospitales, 08009 Barcelona, Spain; 6 Dentistry Department, Universidad de Antofagasta, Antofagasta 1270300, Chile; maria.campillay.riquelme@ua.cl; 7 Biomedical Department, Faculty of Health Sciences, Universidad de Antofagasta, Antofagasta 1270300, Chile; 8 Service of Oral and Maxillofacial Surgery, Institut Ferrés Amat, 08021 Barcelona, Spain

**Keywords:** traumatic dentoalveolar injuries, titanium trauma splints, pediatric dentistry

## Abstract

*Background and Objectives*: Traumatic dentoalveolar injuries (TDI) in children often require urgent stabilization using splints. Titanium trauma splints (TTS) represent a practical option; however, pediatric evidence from hospital-based emergency settings remains limited. This study describes the clinical and contextual characteristics of children treated with TTS and explores factors associated with early complications and splint stability. *Materials and Methods*: A retrospective observational cohort study was conducted at a Pediatric Dentistry Service, including children with TDI managed with TTS and followed for a minimum of three months. Clinical records were reviewed to collect demographic, contextual, and clinical variables. Early complications and the need for re-splinting were recorded, and associations between selected variables and outcomes were analyzed. *Results*: Seventy-three patients (64.4% male; mean age 10.29 ± 2.99 years) and 127 traumatized teeth (98.4% permanent) were included. A predominance of school-based injuries was observed (52.1%). The most frequent injury types were subluxation (39.1%), avulsion (26.6%), and extrusion (16.4%). A longer interval between trauma and splint placement was associated with inflammatory root resorption (*p* = 0.011), although this finding should be interpreted with caution given the limited number of events. Mixed-dentition splints showed a higher likelihood of requiring re-splinting (OR = 12.23; 95% CI: 1.18–126.60); however, this estimate was imprecise and should be interpreted as an exploratory signal. Overall, 90.4% of patients completed treatment with a single splint. *Conclusions*: Within the limitations of this retrospective observational cohort, TTS showed satisfactory short-term clinical stability in pediatric traumatic dental injuries. Longer time between trauma and splint placement was associated with inflammatory root resorption, while mixed-dentition splints emerged as a potential signal of increased re-splinting. These findings are exploratory and hypothesis-generating and require confirmation in future studies.

## 1. Introduction

Traumatic dentoalveolar injuries (TDI) represent a common and clinically significant condition in pediatric populations, with reported prevalence ranging from 11.1% to 22.7% depending on the stage of dentition [1,2,3]. These injuries frequently require urgent management in pediatric healthcare settings, where clinical decision-making is often influenced by patient age, cooperation, and the presence of mixed dentition. Beyond their immediate impact on oral structures, TDI can have long-term consequences on function, aesthetics, and psychosocial well-being, particularly in children and adolescents [4,5,6,7].

The management of TDI in pediatric patients presents specific clinical challenges. Immature permanent teeth are especially vulnerable, as trauma may disrupt root development and compromise pulp vitality, thereby affecting long-term dental and craniofacial growth [8,9,10,11,12]. In addition, outcomes are influenced not only by injury severity but also by contextual factors, including time to treatment, caregiver awareness, and access to emergency care [13]. These factors contribute to the variability observed in clinical outcomes following trauma.

Among TDI, avulsion and severe luxation injuries are associated with the highest risk of complications. Current International Association of Dental Traumatology (IADT) guidelines recommend prompt repositioning and stabilization using flexible or semi-rigid splints to support periodontal and pulpal healing [11,14]. The primary objective of splinting is to maintain functional stability while preserving physiological mobility, which is essential for optimal tissue repair. In pediatric patients, this balance may be more difficult to achieve due to anatomical and behavioral factors, as well as the presence of mixed dentition. Various splinting techniques have been described, including composite wire systems, fiber-reinforced splints, and titanium-based meshes [15,16,17,18,19]. Rather than representing a single optimal approach, these methods should be understood within the broader context of clinical applicability, handling characteristics, and adaptability to different clinical scenarios. In particular, the selection and performance of splinting systems in real-world pediatric settings remain influenced by practical considerations such as ease of use, treatment conditions, and patient-related factors.

In this context, the analysis of clinical outcomes following splinting in pediatric trauma cases managed as emergencies in hospital settings may provide valuable insights into the management of these injuries. However, despite the widespread clinical use of flexible splinting systems, there is limited real-world pediatric evidence from hospital-based emergency care specifically addressing the performance of TTS and factors associated with early clinical outcomes and re-intervention, including the need for re-splinting. This gap is particularly relevant in hospital environments, where treatment conditions and patient-related factors may differ from controlled or ideal clinical scenarios.

Therefore, the aim of the present study was to evaluate early clinical outcomes and to explore factors associated with complications and the need for re-splinting in teeth affected by TDI managed with TTS in a pediatric hospital setting. By focusing on routine clinical conditions, this study seeks to contribute to a better understanding of the challenges and determinants of treatment success in pediatric dental trauma.

## 2. Materials and Methods

### 2.1. Design, Patients, and Inclusion/Exclusion Criteria

A retrospective observational cohort study with short-term follow-up was conducted through the review of clinical records of pediatric patients treated for TDI by splinting in the emergency department of the Pediatric Dentistry Service at Hospital HM Nens (HM Hospitals). The analytical framework was descriptive and hypothesis-generating, exploring associations between predefined clinical variables and early post-traumatic outcomes. The study was conducted in accordance with the Declaration of Helsinki and followed the recommendations of the STROBE statement for observational studies. The study period included initial treatments performed between January 2021 and July 2021, with follow-up evaluations conducted three months after the traumatic event. The protocol was authorized and approved by the Hospital’s Scientific Council and by the Ethics Committee (Act 3/14; Ethics Committee for Research with Medicines of HM, Code: 23.02.2160-GHM).

The following inclusion criteria were established for the study:

- Clinical records of pediatric patients who sustained TDI and required treatment by means of splinting, with a minimum documented three-month follow-up period.
- Availability of complete and relevant clinical information in the medical records required for data extraction.

In addition, the following exclusion criteria were applied:

- Clinical records of patients with documented special healthcare needs.
- Records indicating the presence of motor disorders.
- Cases in which scheduled follow-up visits were not documented in the clinical records.
- Clinical records of patients with a documented history of TDI in the same treated anatomical region.

The exclusion of patients with a documented history of previous TDI in the same anatomical region was intentionally applied to reduce potential confounding associated with pre-existing structural compromise or prior therapeutic interventions. This approach allowed the present analysis to focus on primary injury events within a more controlled clinical framework. In addition, patients with special healthcare needs or motor disorders were not included, as their management often requires specific clinical adaptations, including modified treatment timing, procedural adjustments, and, in some cases, general anesthesia. These factors may introduce variability that is not directly comparable with the standardized treatment protocol applied in this study. Although this criterion may theoretically limit external validity, it was defined a priori to ensure greater procedural consistency and comparability within the analyzed cohort.

### 2.2. Treatment Selected, Data Processing and Variables Included in the Study

The splint system used was the titanium trauma splint (TTS^®^, Medartis AG, Basel, Switzerland), consisting of a pure titanium mesh with a rhomboidal design featuring openings measuring 1.8 × 2.8 mm and a thickness of 0.2 mm. The material can be easily adapted to the dental arch, facilitating standardized application across patients and reducing operator dependent variability during the splinting procedure.

**Figure 1:**
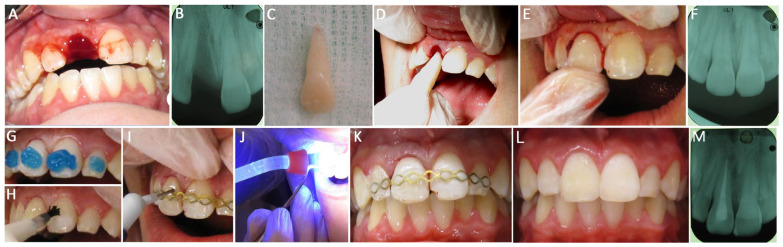
Clinical case of an 11-year-old male patient treated at the Pediatric Dentistry Service of Hospital HM Nens (Barcelona) following a TDI sustained at school, illustrating the treatment sequence of an avulsed tooth stabilized using TTS. (**A**–**C**) Avulsion of tooth 1.1 with a minimal coronal fracture at the incisal edge. Tooth 2.1 also presented an enamel fracture involving the mesioincisal angle. The tooth remained extraoral for more than 60 min and was transported in moist gauze; TTS stabilization was performed on the same day as the TDI. Following local anesthesia, a preoperative radiograph excluded alveolar and root involvement, and the tissues surrounding the socket were gently irrigated with sterile saline, avoiding manipulation of the socket. (**D**,**E**) After surface management according to IADT recommendations, the tooth was repositioned using digital pressure. (**F**) Radiographic confirmation of tooth repositioning. (**G**,**H**) Surface cleaning, acid etching at fixation sites, and adhesive application. (**I**,**J**) TTS placement using composite resin with light curing. (**K**) Occlusal assessment. (**L**,**M**) Clinical and radiographic outcome following TTS removal at the two-week follow-up. The soft tissues showed favorable healing conditions. Teeth 1.1 and 2.1 were restored at the incisal edge. The radiograph demonstrates the avulsed tooth correctly positioned within its alveolus. Endodontic treatment was initiated 1 week after splint placement. Calcium hydroxide [Ca(OH)_2_] was used as an intracanal medicament for 2 weeks, after which the canal was obturated. Images were published with written parental consent and minor assent and were fully anonymized. The case was not part of the study cohort.

The splinting procedure followed a standardized protocol including local anesthesia, cleaning and disinfection of the affected area, repositioning of displaced teeth and supporting tissues when required, and adaptation of the TTS segment. Tooth position was verified radiographically, followed by occlusal assessment and preparation of the enamel surfaces at fixation sites using 37% phosphoric acid. A two-step etch-and-rinse adhesive system (Tetric^®^ N-Bond, Ivoclar, Zurich, Switzerland) and a composite resin (Tetric^®^ N-Flow, Ivoclar) were used for fixation. Light-curing was performed using an LED unit (≈430–480 nm, ≥1000 mW/cm^2^) for 10–20 s for the adhesive and 20 s per increment of composite resin. Final radiographic and occlusal controls were performed after splint placement. A representative clinical sequence is illustrated in Figure 1.

The duration of titanium semi-rigid splinting was determined in accordance with the recommendations of the International Association of Dental Traumatology, based on the type of injury [11,20]. In general, splints were maintained for approximately two weeks in cases of subluxation, extrusion, and avulsion, and for four weeks in cases of lateral luxation and root fracture injuries, unless specific clinical circumstances required minor adjustments. Postoperative instructions followed current IADT recommendations and included advice on maintaining careful oral hygiene with a soft toothbrush, the use of chlorhexidine rinses when indicated, adherence to a soft diet, avoidance of contact sports, and the use of analgesics as required. Patients were scheduled for follow-up evaluations one week after splint placement, at the time of splint removal, and again three months after the traumatic incident, although minor variations in attendance timing may occur due to routine clinical scheduling adjustments or caregiver-related circumstances inherent to real-world hospital follow-up.

All splinting procedures were performed by four pediatric dentists from the Pediatric Dentistry Service of Hospital HM Nens, each with a minimum of three years of institutional clinical experience in pediatric trauma care. The application of TTS followed a standardized institutional protocol aligned with current IADT guidelines and the manufacturer’s technical recommendations. Prior to the study period, all operators had at least one year of experience using this splinting system. The use of a uniform protocol and experienced operators was intended to minimize inter-operator variability.

Each patient was assigned a unique identification code to ensure data confidentiality. Patient management and data recording were performed by four calibrated dentists from the Pediatric Dentistry Service at Hospital HM Nens using a standardized Case Report Form (CRF) in accordance with institutional clinical protocols. Information recorded in the CRF was cross-verified against original clinical records, including medical histories and routine clinical notes. Non-clinical variables, such as the place and circumstances of injury, were extracted from caregiver-reported information documented during standard anamnesis at the time of care; no additional interviews were conducted for research purposes. Given the retrospective design and the use of anonymized data, informed consent was waived by the institutional ethics committee.

A structured data collection form was used to systematically record clinical variables, including the type of trauma according to Andreasen et al. [21], as adapted by Petti et al. [1], number of affected and splinted teeth, type of dentition involved (primary and/or permanent), presence of soft-tissue injury, need for pulp therapy, and post-traumatic complications. Contextual variables included patient age, gender, date of trauma, and location of injury (home, school, or sports setting). Time to splint placement was recorded from clinical files and categorized in calendar days (0 = same-day care; 1 day; 2 days; or more). Due to emergency hospital workflows, including triage, interdepartmental referral, and caregiver-dependent access, minute-based timing was not consistently available; thus, the “0-day” category reflects same-day care rather than immediate intervention.

In cases with follow-up beyond three months, care was continued within the same service when necessary, particularly in the presence of early or medium-term complications, or was referred to the corresponding treating dentist or to community-based services; however, for the purposes of this study, only data within the predefined three-month observational period were included, as this corresponds to the standard minimum follow-up protocol for all TDI cases requiring splinting in the Pediatric Dentistry Service at the hospital. Accordingly, only patients with a minimum follow-up of three months were included in the analysis, which may introduce a potential risk of selection bias.

### 2.3. Outcome Assessment

Post-traumatic complications were identified using standardized clinical and radiographic criteria routinely applied in pediatric dental trauma care, consistent with current IADT recommendations and incorporated into institutional clinical protocols. Root resorption, pulp necrosis, and infectious complications were diagnosed based on predefined clinical findings and radiographic features, including the presence of radiolucent areas along the root surface, loss of lamina dura continuity, periapical radiolucency, or other signs consistent with inflammatory or replacement resorption.

Re-splinting was defined as the need for premature replacement or reapplication of the splint before the planned removal date due to mechanical failure. Mechanical failure was considered present in cases of partial or complete debonding, loss of splint integrity, or clinically relevant instability compromising adequate tooth stabilization. Caregivers were instructed to return immediately if splint loosening or detachment was observed.

Due to the retrospective nature of the study, outcome assessment was based on clinical records and radiographic documentation, and no blinding of evaluators was performed.

### 2.4. Statistical Analysis of the Information

For analytical purposes, two primary outcomes were defined: (1) development of post-traumatic complications during the three-month follow-up period and (2) need for re-splinting prior to scheduled removal. Variables examined in relation to these outcomes included time elapsed between trauma and splint placement, type of dentition involved in the splinting unit (permanent-only versus mixed-dentition), patient age, gender, number of affected teeth, and presence of soft-tissue injury. Continuous variables were analyzed descriptively for the overall sample and stratified by gender. Group comparisons for continuous variables were performed using the Mann–Whitney U test. Associations between categorical variables were evaluated using contingency tables and analyzed with chi-square or Fisher’s exact tests when expected cell counts were low. Odds ratios (ORs) with corresponding 95% confidence intervals (CIs) were calculated to estimate the magnitude of associations. All statistical analyses were conducted using SPSS version 25 (IBM Corp., Chicago, IL, USA). Post hoc power calculations were performed using G*Power version 3.1.9.6 (Heinrich-Heine-Universität Düsseldorf, Düsseldorf, Germany) to contextualize the study’s capacity to detect associations given the observed sample size and number of events. These calculations were interpreted descriptively and not as confirmatory measures of statistical validity. A *p*-value < 0.05 was considered statistically significant. Because splinting was applied as a single stabilization unit per patient, inferential analyses were primarily conducted at the patient level. Although tooth-level events were recorded descriptively, no hierarchical or cluster-adjusted models were implemented. This approach was adopted considering the expected sample size and cluster structure, which were not suitable for stable estimation using multilevel models. As a result, potential intra-patient correlation could not be formally accounted for and may have influenced the precision of the estimated associations.

Given the retrospective observational design, potential sources of bias were considered. To minimize information bias, data were extracted using standardized CRFs and cross-verified against original clinical records. All patients were treated within the same institutional setting under uniform trauma management protocols aligned with IADT recommendations, thereby reducing variability in clinical procedures and operator-related differences. Inclusion criteria required documented follow-up of at least three months to ensure consistency in outcome assessment.

Although residual confounding cannot be entirely excluded, findings were interpreted cautiously as exploratory associations. In line with the descriptive, hypothesis-generating nature of the study, multivariable regression modeling was not performed, as the study design and expected number of events relative to the variables of interest did not support reliable or stable adjusted estimates. The analytical strategy was therefore designed to explore associations between predefined outcomes and selected clinical and demographic variables, rather than to derive adjusted causal estimates.

## 3. Results

### 3.1. Baseline Demographic and Clinical Characteristics of Pediatric Patients with TDIs Requiring Dental Splinting

**Figure 2:**
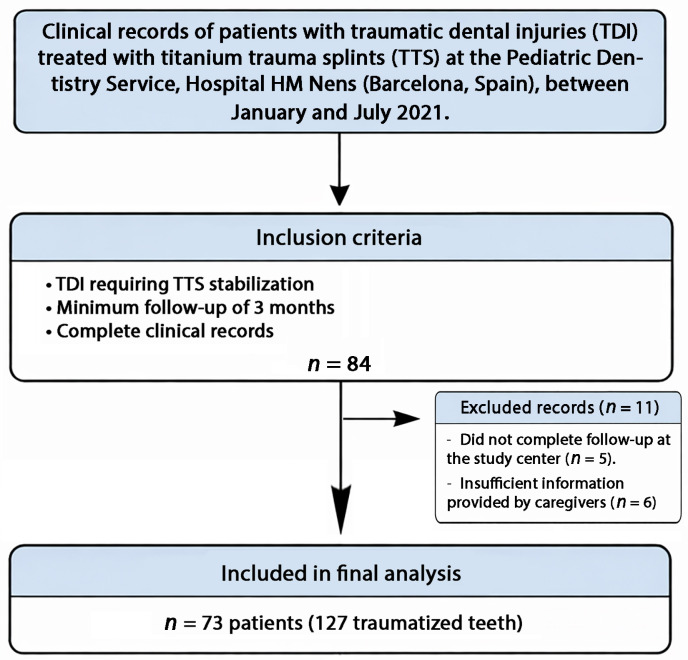
Flow diagram of patient selection and inclusion in the final analysis.

During the study period, 84 patients received TTS at the institution. Of these, 11 were excluded for not meeting the predefined inclusion criteria. The final analytical sample comprised 73 patients (127 teeth), as shown in Figure 2.

The study population was predominantly male, with a mean age of approximately 10 years. Splint placement was mainly performed as same-day care (“0”), although delays of several days were observed in a subset of cases (Table 1). A total of 127 teeth were affected (125 permanent and 2 primary), primarily in the maxillary arch (117 teeth, 92%), with a relatively low number of teeth involved per patient. 

In terms of reimbursement and treatment coverage, three main patient groups were identified in the institutional setting: patients with health insurance coverage, patients covered through school or sports accident insurance, and private patients. In cases involving TDIs sustained during school or sports activities, treatment was frequently covered through accident insurance after institutional reporting procedures.

Regarding the circumstances of injury, a predominance of school-based events was observed, followed by those occurring in the home environment and sports facilities. A variation between male and female patients was observed, as TDIs in males occurred more frequently at school, whereas in females they occurred more often at home (Table 1). Soft-tissue involvement was present in 53.4% of patients.

In total, 278 teeth were splinted using the TTS to stabilize the traumatized units. Female patients showed a slightly higher number of splinted teeth compared to males (Table 1). Most splints were placed exclusively in the maxillary arch, accounting for 67 cases (91.8%). Mandibular-only splints were uncommon, observed in 4 patients (5.5%). Splints involving both arches were the least frequent, occurring in only 2 cases (2.7%).

Overall, as the study population consisted of patients requiring TTS, the injury profile was characterized by a predominance of moderate-to-high-severity cases. Subluxation emerged as the most frequently recorded injury type, followed by avulsion and extrusion, whereas lateral luxation and root fractures were less frequent, and intrusion represented the least commonly reported injury in the sample. Initial trauma management and TTS placement generally required approximately 30–75 min, depending on TDI complexity and the need for repositioning maneuvers. TTS stabilization periods ranged from 14 to 30 days according to injury severity and IADT recommendations. Shorter stabilization periods were commonly used for avulsion and extrusion injuries (14 days), whereas longer periods were required in selected cases involving lateral luxation, alveolar fractures, root fractures, or surgically repositioned intrusion injuries (28–30 days). No cervical root fractures or complex alveolar fractures requiring extended stabilization periods beyond these ranges were observed in the present cohort.

Considering the biological relevance of avulsion injuries, additional descriptive data were collected. Among the 34 avulsed teeth included in this cohort, milk was the most frequently reported storage medium (*n* = 22), followed by saline solution (*n* = 4), moist gauze (*n* = 3), and water (*n* = 5). Extra-oral dry time was not consistently documented in the clinical records and could not be reliably determined due to the retrospective design and reliance on caregiver-reported information. Table 2 summarizes the distribution of injury types according to affected teeth, patient gender, and age groups.

In this cohort, most patients completed treatment without requiring re-splinting during the observation period. No splint-related soft-tissue lesions or patient-reported discomfort were documented in the clinical records. Splint placement allowed access for pulpal interventions when required and did not interfere with routine oral hygiene, while physiological mobility of the injured teeth was maintained throughout the stabilization period.

Due to pulpal involvement, 33 permanent teeth required pulp treatment (26.4% of all permanent teeth). These cases were predominantly associated with maxillary incisors, particularly maxillary central incisors (27 teeth; 21.6%), followed by maxillary lateral incisors (5 teeth; 4%), with only one mandibular central incisor affected (0.8%). Additionally, six teeth required induction of apical development (4.8% of all permanent teeth), all corresponding to maxillary incisors.

### 3.2. Post-TDI Complications and Related Clinical Variables

#### 3.2.1. Descriptive Overview of Complications

A limited proportion of affected teeth developed complications during follow-up. Isolated cases of phlegmon, pulpal necrosis, and root resorption following TDI treatment were documented (a total of 10 cases). Table 3 provides a descriptive overview of the identified complications and the clinical variables associated with these cases.

#### 3.2.2. Splint Stability and Need for Re-Intervention

Evaluation during follow-up showed overall stability of the splinting system. Most patients completed treatment with a single splint, whereas seven required re-splinting prior to removal. Among these seven cases, two required a new splint following extraction of a traumatized tooth due to complications, and one underwent premature removal because of a new TDI. After excluding these events, only four patients required a second splinting procedure due to unexplained deterioration or debonding (Table 4).

#### 3.2.3. Inferential Analysis of Variables Associated with Complications

Patients who developed complications exhibited a longer mean interval between the TDI and splint placement compared with those without complications, although this difference was not statistically significant (Table 5). However, as shown in Table 3, root resorption occurred in cases of avulsion and extrusion; two of these patients received care as same-day care (“0”), whereas the remaining cases were treated several days later. When comparing the time elapsed between trauma and splint placement in patients who developed root resorption versus those who did not, a statistically significant difference was observed (*p* = 0.011). However, this estimate should be interpreted with caution, as the statistical power was limited (approximately 45–55%) due to the small number of events, indicating that the analysis was primarily able to detect relatively large effect sizes. In this context, the possibility of imprecision and an increased risk of type I error cannot be excluded.

The mean number of affected teeth was higher in patients with complications than in those without, although this difference was not statistically significant. The distribution of complications was similar between genders. Soft-tissue injuries were more frequent among patients with complications, but this trend did not reach statistical significance, and the corresponding odds ratio should be interpreted cautiously due to imprecision.

Overall, aside from the observed difference related to delayed splint placement and root resorption, the remaining variables evaluated did not demonstrate statistically significant differences and should be interpreted with caution. Given the limited number of events, no adjusted analyses were performed, and potential confounding by factors such as injury severity or trauma type (e.g., avulsion vs. non-avulsion) cannot be excluded. A descriptive review by injury type indicated a higher proportion of complications in avulsion cases; however, this observation should be considered a descriptive trend within the cohort.

#### 3.2.4. Inferential Analysis of Variables Associated with Re-Splinting

Re-splinting procedures were more frequently observed in cases involving both permanent and primary teeth (Table 4). Among all splinting procedures, 17 involved mixed dentition (23.3%). A higher frequency of re-splinting was observed among splints involving both permanent and primary teeth compared with those restricted to permanent teeth (OR = 12.23; 95% CI: 1.18–126.60). However, this estimate was highly imprecise, as reflected by the wide confidence interval, and should therefore be interpreted with caution. The analysis excluded two cases requiring re-splinting due to tooth extraction following complications and one case with a new traumatic dental injury during treatment. Given the small number of events, statistical power was limited, increasing the risk of type I error and restricting the ability to detect anything other than large effects. Accordingly, this finding should be interpreted as an exploratory, hypothesis-generating signal rather than as evidence of a robust association.

## 4. Discussion

Dentoalveolar trauma represents a frequent clinical challenge in pediatric populations, with a higher prevalence among males and a distribution influenced by age and environmental context [1,22]. In the primary dentition, the home remains the predominant setting (43–88%) [23,24,25], while school becomes a major site of injury with increasing age, and adolescence is more commonly associated with recreational and sports-related environments [25,26,27]. In the present cohort, these patterns were preserved despite the sample being composed predominantly of moderate-to-severe TDI cases, with an even greater predominance of male patients observed. Regarding injury setting, school was the most frequent location overall, whereas a higher proportion of injuries in females occurred at home. The mean age (10.29 years) falls within the peak prevalence range described by Bastone et al. [22], consistent with the epidemiological patterns reported by Glendor [2,3].

The time elapsed between traumatic injury and professional care remains a key prognostic factor in TDIs and is often poorly understood among caregivers and first responders in some communities [13,28]. According to IADT guidelines, prompt intervention is particularly critical in avulsion injuries, where replantation within 60 min favors periodontal and pulpal outcomes, while delays increase the risk of inflammatory resorption, ankylosis, and impaired revascularization [11,29]. In the present study, the time between TDI and splint placement ranged from same-day care to 10 days, with most patients receiving treatment within the first 24 h. A limited number of complications were observed, including cases of root resorption that showed a statistically significant association with longer treatment delays, although this finding should be interpreted cautiously given the moderate statistical power. Rather than reflecting an effect attributable to the splinting system itself, this observation is consistent with the well-established biological impact of delayed intervention on periodontal ligament viability and pulpal status [10,11,29,30]. These findings reinforce the importance of timely management, particularly in severe injuries, while remaining within an exploratory interpretative framework. In hospital-based pediatric settings, however, treatment timing is often influenced by triage protocols, referral pathways, and caregiver-dependent access to care, and is therefore commonly recorded in calendar days rather than precise intervals, reflecting real-world emergency workflows [25,31]. This context should be considered by caregivers, clinicians, and health policy frameworks, as hospital-based trauma management frequently extends beyond tooth-specific considerations and may differ from guideline recommendations primarily focused on TDI-centered scenarios.

Splinting remains a fundamental component of emergency management when dental mobility compromises comfort, function, or oral hygiene. Current guidelines recommend flexible splints for most luxation injuries, whereas more rigid systems are reserved for alveolar fractures and selected root fractures [11]. However, the available evidence indicates that splint characteristics exert only a limited influence on biological healing. Studies by Kahler et al. [16,31] and subsequent investigations [32,33] have shown that outcomes are primarily determined by injury severity and periodontal ligament damage rather than by the type or duration of splinting, provided that guideline-based protocols are followed. In this context, avulsion injuries in the present cohort were descriptively associated with a higher frequency of complications, consistent with their recognized biological severity [10,11,20]. Given the limited number of events and heterogeneity of injuries, these observations should be interpreted as descriptive trends within an exploratory framework. Overall, the use of short-term flexible splints remains a biologically grounded and guideline-supported approach.

TTS are low-rigidity, flexible systems that align with these biomechanical principles, allowing for near-physiological mobility and favorable handling characteristics [14,31]. Experimental and clinical studies have reported adequate comfort, minimal interference with function, and good patient acceptance, while the titanium mesh design facilitates adaptation and access for oral hygiene [17,18,34]. In this context, effective plaque control is critical for periodontal healing following trauma, and structured hygiene protocols have been shown to improve clinical outcomes during splinting [11,19,20,34,35,36]. In vitro evidence further suggests that TTS may exhibit reduced biofilm accumulation compared with other splinting systems, potentially offering an additional advantage for periodontal stabilization [35,37]. Despite these advantages, practical considerations include higher cost, single-use requirements, and the potential for plaque accumulation or soft-tissue irritation if bonding is suboptimal. Moreover, the available evidence remains limited, and current data indicate that TTS provides stabilization within the range reported for other flexible splinting systems rather than demonstrating superiority [17,18,19,37]. The findings of the present study are consistent with this perspective, supporting the clinical applicability of TTS under routine conditions without allowing conclusions regarding comparative effectiveness. Notably, the present study provides clinically relevant information in a context where reports on the use of TTS in hospital-based pediatric TDI management, such as the setting described here, remain scarce in the literature.

Complications such as splint debonding and inflammatory root resorption have been reported at low frequencies and are primarily related to the severity of the initial injury rather than to the splinting system itself [16,38], a pattern consistent with the observations of the present study. In hospital-based pediatric settings, where trauma management is typically preceded by broader medical assessment and triage, treatment conditions and timing may further influence these outcomes. Within this context, and in line with current evidence indicating no clear superiority among flexible splinting systems when guideline-based protocols are followed [16,31], the findings of this study should be interpreted as exploratory. Further well-designed clinical studies are required to better define the long-term performance and prognostic value of TTS within real-world hospital-based trauma management, where treatment conditions and timing may differ from ideal guideline scenarios.

In the present study, splints involving both permanent and primary teeth emerged as a potential signal of increased re-splinting, with mixed dentition splints showing more than twelve times the odds compared with splints restricted to permanent teeth. However, the low number of events, wide confidence interval, and moderate statistical power substantially limit the inferential strength of this finding, which should be interpreted as exploratory and hypothesis generating. This observation aligns with previous reports describing technical challenges in mixed dentition, particularly in cases involving extended splint spans or partially erupted teeth [39]. Although flexible splinting systems provide adequate stability under ideal conditions [40], clinical scenarios involving primary teeth introduce additional variables such as exfoliative stage, reduced crown height, enamel characteristics, and patient related factors that may compromise bonding reliability. Accordingly, while flexible splinting remains biologically appropriate, particular caution may be warranted when primary teeth are used as abutments in real world conditions, pending confirmation from larger studies [41].

### Limitations

This study presents several limitations. It is based on a moderate-sized single-center cohort with a limited number of events (i.e., adverse clinical outcomes such as complications and re-splinting), which may limit its generalizability and statistical power, particularly for evaluating associations such as treatment delay and complications. In line with the study design and expected data structure, multivariable adjustment and cluster-adjusted analyses were not applied, as the available sample and event distribution did not support stable or reliable estimation and would increase the risk of overfitting in multivariable models. Consequently, residual confounding cannot be excluded, and potential intra-patient correlation could not be formally accounted for, which is unlikely to have substantially influenced the results, although a potential minor effect on the precision of the estimated associations cannot be excluded.

The most novel finding, namely the potential signal of increased re-splinting in cases involving primary teeth, should therefore be interpreted with caution, as the limited number of cases may not fully capture variability in mixed dentition. Relevant clinical factors such as exfoliative stage, crown morphology, patient cooperation, and root development could not be adequately controlled and may have influenced outcomes. In addition, heterogeneity of injury types, variability in time to treatment, and incomplete documentation of key variables such as extra-alveolar dry time and storage conditions further constrain inference, despite their recognized prognostic relevance.

Importantly, this study was conducted in a hospital-based setting where trauma management often follows broader medical assessment and triage and does not always prioritize immediate tooth-specific intervention. Consequently, treatment conditions and timing reflect routine clinical workflows, and variable control is inherently limited to routinely collected clinical data, which may introduce information bias inherent to retrospective record-based studies, particularly as key variables such as time to injury and initial management were in part based on caregiver-reported information and subsequently recorded in clinical files. The absence of a comparison group using alternative splinting systems precludes attribution of outcomes specifically to TTS and limits comparative conclusions. Moreover, the three-month follow-up restricts the analysis to early post-traumatic evolution and does not allow assessment of late complications such as resorption or ankylosis, which are primarily influenced by injury severity rather than splint type [9,11,16,20,31]. Finally, inclusion criteria requiring documented follow-up may have introduced selection bias, and patient-related behavioral factors such as oral hygiene and adherence to instructions were not systematically recorded. Accordingly, these findings should be interpreted within an exploratory, real-world clinical framework, and further multicenter studies with extended follow-up are needed to confirm the observed signals, particularly regarding splinting in mixed dentition. The applicability of these findings to patients with special healthcare needs or motor disorders may be limited, as these populations often require specific clinical adaptations; however, no patients with these characteristics were managed for TDI in the study setting during the study period, and therefore this exclusion criterion was not applied in practice.

## 5. Conclusions

Within the limitations of this retrospective observational cohort, TTS showed satisfactory short-term clinical stability in the management of traumatic dental injuries in pediatric patients. Consistent with current evidence, post-traumatic complications appeared to be more closely related to injury severity and treatment delay than to the splinting system itself. Importantly, these findings arise from a hospital-based setting, where trauma management often follows broader medical priorities that may influence treatment timing. Longer time between trauma and splint placement was associated with inflammatory root resorption, while splints involving both permanent and primary teeth emerged as a potential signal of increased re-splinting. Given the limited number of events, these findings should be interpreted as exploratory and hypothesis-generating and require confirmation in future controlled studies.

## Figures and Tables

**Table 1:** Demographic and key clinical characteristics of patients treated with splints.

Variable	Total (*n* = 73)	Male (*n* = 47)	Female (*n* = 26)	*p*-Value
Mean age (min–max)	10.29 (6–18)	10.66 (6–17)	9.62 (7–18)	0.154 ^b^
Mean time elapsed until treatment in days (min–max) ^a^	0.9 (0–10)	1.09 (0–10)	0.62 (0–8)	0.423 ^b^
Mean number of affected teeth (min–max)	1.74 (1–5)	1.74 (1–5)	1.73 (1–5)	0.708 ^b^
Mean number of splinted teeth (min–max)	3.81 (2–9)	3.66 (2–8)	4.08 (2–9)	0.164 ^b^
Most frequent trauma location	School (52.1%)	School (55.3%)	Home (50%)	0.136 ^c^
	Home (36.9%)	Home (29.8%)	School (46.2%)	
	Sports facilities (10.9%)	Sports facilities (14.9%)	Sports facilities (3.9%)	

^a^ = “0” represents “same-day care”. ^b^ = Mann–Whitney U test. ^c^ = Chi-square test.

**Table 2:** Distribution of injury types by affected teeth, patient gender, and age group.

Type of Injury (*n*/%)	Affected Permanent Teeth According to FDI Notation (*n*)		Affected Primary Teeth According to FDI Notation (*n*)		Female Cases	Male Cases	Age Groups		
	Maxillary	Mandibular	Maxillary	Mandibular			≤8 Years	>8–11 Years	>11–18 Years
Avulsion (34/26.6%)	21 (12), 11 (10), 12 (5), 22 (2)	31 (1), 41 (1)	52 (1), 61 (1), 63 (1)	0	18	16	12	13	9
Subluxation (49/39.1%)	21 (18), 11(18), 22 (6), 12(2),	31 (2), 41 (2), 42 (1)	0	0	18	31	23	14	12
Extrusion (21/16.4%)	11 (9), 21 (8), 22 (1), 12 (1)	31 (2)	0	0	3	18	8	6	7
Root fracture (6/4.7%)	21 (3), 11 (3)	0	0	0	2	4	2	2	2
Lateral luxation (13/10.2%)	11 (6), 21 (5), 12 (1)	32 (1),	0	0	5	8	2	4	7
Intrusion (4/3.1%)	21 (2), 11 (1), 12 (1)	0	0	0	0	4	3	0	1

FDI = Fédération Dentaire Internationale (two-digit tooth notation system).

**Table 3:** Complications and related clinical variables in splinted teeth following TDI *.

Complication Type ^a^	Teeth Exhibiting Complications (FDI Notation)	Age (Years)	Gender	Days from TDI to Splint ^b^	Total Teeth Affected by TDI	Injury Type (TDI Classification)	Endodontic Treatment Performed During Follow-Up	Teeth Requiring Extraction	Soft Tissue Involvement
Phlegmon	12	7	Female	0	1	Avulsion	No	No	Yes
Phlegmon	11	7	Male	0	1	Subluxation	No	No	No
Phlegmon	11; 12; 21	8	Male	0	3	Avulsion	No	No	Yes
Necrosis	21	14	Female	0	1	Lateral luxation	Yes	No	No
Root resorption	11	7	Female	8	5	Avulsion	Yes	No	Yes
Root resorption	11	7	Female	1	1	Avulsion	Yes	No	Yes
Phlegmon plus root resorption.	21	8	Male	5	2	Avulsion	No	Yes	Yes
Root resorption	21	10	Male	0	2	Extrusion	No	No	No
Root resorption	21	11	Male	10	4	Extrusion	No	No	Yes
Root resorption	21	18	Female	0	2	Avulsion	Yes	No	Yes

^a^ = The ten cases with complications are presented along with their relationship to the other variables. ^b^ = “0” represents “same-day care”. FDI = Fédération Dentaire Internationale (two-digit tooth notation system). * Complications observed during the three-month follow-up.

**Table 4:** Splint stability, re-splinting events, and associated dentition type during follow-up.

Splint Outcome		*n* (%)	Mixed Dentition (*n*)	Permanent Dentition (*n*)	*p* Value	OR (95% CI)
Completed with a single TTS		66 (90.4%) *	13	53	0.074 ^a^	12.23 (1.18–126.60)
Required re-splinting prior to removal	Second splinting procedure due to unexplained deterioration or debonding	4 (5.5%) *	3	1		
	Premature removal due to a new TDI	1 (1.4%)	0	1	---	---
	New splint following extraction of a traumatized tooth due to complications	2 (2.7%)	1	1	---	---

^a^ = Fisher’s exact test. OR = odds ratio. CI = confidence interval. * Fisher’s exact test and OR were calculated only for re-splinting due to unexplained deterioration or debonding.

**Table 5:** Association of observed complications with other variables.

Variables	Patients with Post-Treatment Complications (*n* = 10)	Patients Without Post-Treatment Complications (*n* = 63) ^c^	*p*-Value	OR (95% IC)
Time between TDI and Splint Placement	mean = 2.43 (0–10 days)	mean = 0.68 (0–8 days)	0.239 ^a^	—
Number of teeth affected by TDI	mean = 2.21 (1–5 teeth)	mean = 1.67 (1–5 teeth)	0.259 ^a^	—
Gender	Female = 5	Female = 21	0.314 ^b^	0.50 (0.13–1.95)
	Male = 5	Male = 42		
Soft tissue injuries	Observed = 7	Observed = 32	0.320 ^b^	2.26 (0.52– 9.77)
	Not observed = 3	Not observed = 31		

^a^ = Mann–Whitney U test. ^b^ = Fisher’s exact test. ^c^ = “0” represents “same-day care”. OR = odds ratio. CI = confidence interval.

## Data Availability

The data associated with this study consist of patient information archived at the institution where the patients were treated and are not publicly available due to privacy and ethical policies.
